# MicroRNAs in ascending thoracic aortic aneurysms

**DOI:** 10.1042/BSR20200218

**Published:** 2020-07-27

**Authors:** Areti Moushi, Nir Pillar, Anna Keravnou, Marinos Soteriou, Noam Shomron, Marios A. Cariolou, Evy Bashiardes

**Affiliations:** 1 Cyprus School of Molecular Medicine, The Cyprus Institute of Neurology and Genetics, Nicosia, Cyprus; 2 Sackler Faculty of Medicine, Tel Aviv University, Tel Aviv 69978, Israel; 3 Department of Cardiovascular Genetics and The Laboratory of Forensic Genetics, The Cyprus Institute of Neurology and Genetics, Nicosia, Cyprus; 4 Department of Cardiology and Cardiovascular Surgery, American Medical Centre, Nicosia, Cyprus

**Keywords:** biomarkers, microRNAs, plasma samples, thoracic aneurysms

## Abstract

Thoracic Aortic Aneurysm (TAA) is characterized by the dilation of the aorta and is fatal if not diagnosed and treated appropriately. The underlying genetic mechanisms have not been completely delineated, so better knowledge of the physiopathology of TAAs is needed to improve detection and therapy. MicroRNAs (miRNAs) regulate gene expression post-transcriptionally and are known to be involved in cardiovascular diseases (CVDs). The current study aimed to identify miRNAs that can be used as possible biomarkers for the early diagnosis of patients with ascending TAAs (ATAAs). MiRNA expression was profiled by NanoString nCounter technology using 12 samples including tissue and pre- and post-surgical plasma from ATAA patients. Four miRNAs were selected and further validated by real time polymerase chain reaction (RT-PCR) in 22 plasma samples from which three miRNAs (hsa-miR140-5p, hsa-miR-191-5p and hsa-miR-214-3p) showed significant expression level differences between the two types of plasma samples. Further analyses of the corresponding predicted target genes by these miRNAs, revealed two genes (Myotubularin-related protein 4 (*MTMR4*) and Phosphatase 1 catalytic subunit β (*PPP1CB*)) whose expression was inversely correlated with the expression of their respective miRNAs. Overall, in this pilot study, we identified three miRNAs that might serve as potential biomarkers and therapeutic targets in ATAA.

## Introduction

Thoracic aortic aneurysm (TAA) may be defined as a localized or diffuse dilatation of the aorta to at least a 50 percent increase in diameter compared with the expected normal diameter. The aortic root, ascending aorta, aortic arch, or descending aorta may be affected and are classified accordingly [1]. Sixty percent of TAAs involve the aortic root and/or ascending aorta, 40% involve the descending aorta, 10% involve the arch, and 10% involve the thoracoabdominal aorta [2]. The etiology, natural history, and treatment of thoracic aneurysms differ for each of these segments. Ascending TAAs (ATAAs) most often result from cystic medial degeneration, which appears histologically as smooth muscle cell dropout and elastic fiber degeneration. Medial degeneration leads to weakening of the aortic wall, which in turn results in aortic dilatation and aneurysm formation [2]. Cystic medial degeneration occurs normally to some extent with aging, but the process is accelerated by hypertension [3]. ATAAs can either be sporadic or genetically inherited. In younger patients, it is more often associated with a genetic predisposition that can be familial [4] or related to defined genetic disorders such as Marfan syndrome.

The natural history of TAA is one of the slow expansion with a progressive increase in the risk of aortic dissection (AD) at larger aortic sizes. The slow expansion of TAA means that most patients with TAA are asymptomatic and many patients will succumb to other disease processes without being aware that TAA is present. Aneurysms that do produce symptoms are typically very large and at risk for rupture, which is associated with high rates of morbidity and mortality. Death from aneurysmal rupture is one of the fifteen leading causes of deaths in most series. According to the Centers for Disease Control and Prevention, the incidence of ATAA is estimated to be approximately 10 per 100000 person-years [5].

Despite their diverse etiologies, all TAAs are prone to two lethal acute events: AD and aortic rupture. In AD, blood is diverted from its usual location within the lumen of the aorta into a false lumen within the media through a tear in the intima. AD is a catastrophic event in which the layers of the aortic wall split apart. An aortic rupture includes a complete tear or transection through all three layers of the aorta, resulting in massive internal hemorrhage [6]. Eleftheriades et al., 2005 [7], have described the detection of TAAs as essential due to the lethality of the disease, as well as difficult as approximately 95% of TAAs patients are asymptomatic before presented with a devastating fatal or near-fatal dissection or rupture. The mortality is said to be 1% per hour once AD has occurred. Symptomatic patients or those that have complications related to the aneurysm should undergo surgical repair. The mortality of elective surgical repair of ascending aortic aneurysms in large centers is 3–5% [2]. Conservative management of asymptomatic TAA aims to lessen stress on the aorta and limit further aortic expansion. Asymptomatic patients who do not meet criteria for repair also require ongoing aneurysm observation [1,8,9]. Screening for asymptomatic TAAs before an event occurs is of great clinical importance. However, there are no standard screening programs for TAAs. Consequently, TAAs are commonly detected incidentally by contrast-enhanced CT scanning and MR angiography. It is critically important to detect the aneurysm before it reaches dangerous diameters, as surgical treatment is safest and most effective on an elective basis. These detection modalities are not safe or cost-effective as screening methods for the general population. Therefore, better screening methods are warranted for TAAs which are non-invasive, cheap, safe, and widely available. A biomarker is a molecular indicator of a biological process or state and in clinical application, biomarkers are reflective of the pathogenic activity of a disease. A biomarker must be able to measure objectively and quantitatively. The quantity of the biomarker can correlate with the type, risk, or severity of disease. Therefore, ideal biomarkers should be able to predict aneurysm formation well before dilatation, can be detected via imaging techniques, and may also provide information on how close an aneurysm is to dissection or rupture. While a number of biomarkers of aortic aneurysms have been identified and shown promise, they have not led to any clinically utilized testing programs.

MicroRNAs (miRNAs) are a group of highly conserved, small noncoding RNAs 21–25 nucleotides long, which usually negatively modulate gene expression at the post-transcriptional level by incomplete or complete complementary binding to target sequences within the 3′ untranslated regions (UTRs) of mRNA [10]. Therefore, an increased expression of a specific miRNA leads to decreased expression of its target genes and conversely [11].

MiRNAs have been discovered to be important regulators for several diseases, therefore, they can also be significant biomarkers in the detection of diseases, since they have several characteristics of an ideal biomarker [12]. MiRNAs are present in several body fluids which can be collected without or via minimally invasive methods, such as blood, saliva, and urine. They are stable in blood plasma and can withstand extreme conditions such as high pH and they can be stored for a long time under appropriate conditions without their biological integrity being affected [13]. They are differentially expressed in different tissues making them specific for each tissue, which is another significant characteristic for a biomarker [14]. In several studies, it was identified that the expression of miRNA in diseased samples compared with healthy controls is different, therefore these small molecules can be used for the detection of a condition or the progression of the disease [15,16]. The aberrant expression of miRNAs has also been linked to cardiovascular diseases (CVDs) and is likely that they also play a key role in aneurysm formation [17–20]. Studies have already investigated and identified miRNAs which are differentially expressed in aneurysmal sample [18,20–25]. These results show promise for miRNA-based biomarkers that can be used to detect and screen for TAAs. Additionally, by studying both the relevant genes and miRNAs in conjunction with the intermodulation between them may clarify the molecular mechanism of the disease and provide reliable molecular targets for detection also leading to prevention and treatment.

The current study was divided into two phases. During the primary phase, the aim was to identify miRNAs with significantly different expression levels between plasma samples from patients, pre- and post-operatively. The secondary phase, aimed to determine whether the predicted target genes of the miRNAs detected, showed a specific pattern of expression in the two types of plasma samples and speculate that their expression changes could be associated with TAAs.

## Materials and methods

### Sample collection

Samples were collected from 11 male and 4 female patients with sporadic ATAAs undergoing surgery for the repair of the aneurysm at The American Medical Centre, Cyprus. Briefly, the ascending sort extending from the sino-tubular junction to the proximal aortic arch was resected. The resected aorta was replaced with a synthetic graft. Any bicuspid or tricuspid valve with moderate to severe aortic valve regurgitation or stenosis were replaced. In cases of AD with aortic valve insufficiency, we proceeded with resuspension of the valve commissures and aortic valve repair.

The tissue samples (T) were taken from the widest region of the ascending aorta at the time of surgery as well as blood samples before (BB) and 7–8 days after (BA) the surgery, for each participant. The criteria used for the selection of these samples was that all patients presented with an aortic diameter ≥ 50 mm and did not have any transfusions during or immediately after the surgery. Patients’ characteristics including, aortic valve type, presence of hypertension, diabetes or hyperlipidemia were recorded (Table 1). Upon initial evaluation, the patients did not have any manifestation of any phenotypic findings (i.e. iris flocculi, livedo reticularis, aortic valve abnormalities) hinting toward connective tissue disorders or aortic pathology. The excised tissue samples were rinsed in saline solution cut and snap-frozen in liquid nitrogen and stored at −80°C. The blood samples were collected in EDTA tubes and steps were followed in order to reduce the issue of hemolysis. When the blood samples were collected, the needle was removed from the syringe and the blood slowly expelled into the EDTA tube. Once the samples were collected, within 20–30 min they were centrifuged and the plasma collected. This involved a double centrifugation at 35 rpm for 10 min, where the plasma was initially removed carefully to prevent carryover of any cell debris and placed into a clean 2-ml tube and then re-centrifuged for further 10 min and carefully transferred to another clean 2-ml tube. The plasma samples were stored in −80°C until needed. The presentstudy was approved by the Cyprus National Bioethics Committee and each patient gave their signed consent.

**Table 1:** Patients characteristics

Characteristics	Primary phase	Secondary phase
	Number (%)	
Gender (Male)	4 (100%)	7/11 (63.6%)
Average age	73.2 ± 9.4	62.5 ± 7
Tricuspid aortic valve	4 (100%)	6/11 (54.5%)
Hypertension	0 (0%)	4/11 (36.4%)
Diabetes	0 (0%)	1/11 (9.1%)
Hyperlipidemia	0 (0%)	4/11 (36.4%)

### Primary phase: investigation of differentially expressed miRNAs between plasma samples

For the primary phase, miRNA expression analysis, a total of 12 samples were used. These were from four male patients with ATAAs undergoing surgery, including the aortic tissue sample and the plasma samples (BB and BA) from each individual.

### Total RNA extraction

For the plasma samples total RNA was extracted using Plasma/Serum RNA Purification Midi Kit (*Norgen Biotek Corp*.) and concentrated and purified further using RNA Clean-up and Micro-Elute Kit (*Norgen Biotek Corp.)*, following the manufacturer’s instructions. From the four tissue samples, a small piece of approximately 5 mg was used where it was initially homogenized and then sonicated to further disrupt the cells of the tissue. Total RNA was isolated, from each sample, using miRCURY RNA Isolation Kit-Tissue (*Exiqon*), in accordance with the manufacturer’s instructions. The A260/280 ratio and the A260/230 ratios for the purified RNA was measured on a Nanodrop 8000 spectrophotometer (Thermo Fisher Scientific). These data are summarized in Supplementary Table S1. To monitor for hemolysis, an RNA sample extracted from a hemolyzed blood sample was used as a control. The level of the erythrocyte specific hsa-miR-451 was compared between this control sample with each of the extracted plasma samples prior to any experimental work undertaken. This hemolysis indicator allowed for the elimination of samples with high expression levels of hsa-miR-451 [26].

### miRNA expression assay

The multiplexed NanoString nCounter Human v3 miRNA expression assay (NanoString Technologies, Seattle, WA, U.S.A.) was used to profile 798 human miRNAs. Samples from four patients were initially used, which included T, BB and (BA) samples after the repair operation. The assay was performed according to the manufacturer’s protocol. Briefly, up to 100 ng of total RNA was used as input material, with 3 μl of the threefold-diluted sample (in the tissue samples only). A specific DNA tag was ligated on to the 3′ end of each mature miRNA, providing an exclusive identification for each miRNA species in the sample. The tagging was performed in a multiplexed ligation reaction utilizing reverse complementary bridge oligonucleotides to dispose the ligation of each miRNA to its designated tag. All hybridization reactions were incubated at 64°C for 18 h. Excess tags were then removed, and the resulting material was hybridized to a panel of fluorescently labeled, bar-coded reporter probes specific to the miRNA of interest. Abundances of miRNAs were quantified with the nCounter Prep Station via counting individual fluorescent barcodes and quantifying target miRNA molecules present in each sample. The count number for each sample was detected and the mean value for each sample type (BB, BA and T) was calculated. Comparisons were made between tissue samples vs plasma from before the operation (T vs BB), tissue samples vs plasma samples from after the operation (T vs BA) and plasma samples before the operation vs plasma sample after the operation (BB vs BA).

### Statistical analysis

All statistical analysis was conducted using R software version 3.2. NanoString data pre-processing and normalization followed by differential expression analysis was performed using DEseq2 package [27] and in-house scripts [28] identifying the mean values from the patients’ samples. The mean value of negative controls was set as the lower threshold for each sample; only miRNAs with at least 50% of their values above the lower threshold were included in the analysis. The false discovery rate method was used to control multiple observations, and the adjusted *P*-value was used for further analysis. Fold-change difference were calculated for each comparison and the *P*-value was determined where *P*-value <0.05 was considered significant [28–30]. The miRNAs which showed significant expression level differences between BB and BA samples were selected for further investigation. MirPath software from Diana tool [31,32], a prediction algorithm designed using databases of experimentally verified miRNA targets, was used in order to identify pathways which were predicted to be significantly (*P*-value <0.05) associated with the eight miRNAs.

### Validation study

The miRNAs that were predicted to be significantly associated with pathways correlated with ATAAs were used for validation purposes on an additional 22 plasma samples (11 BB and 11 BA) by real time polymerase chain reaction (RT-PCR; Table 1).

### cDNA synthesis and RT-PCR

For cDNA synthesis, qScript microRNA cDNA Synthesis Kit (Quanta Biosciences) was used following manufacturer’s instructions. For RT-PCR, mi-real-time Eva Green Master mix (ROX+) (Metabion) was used. For this reaction, 5 μl of the master mix were used, with 1 μl of PCR primer and 4 μl of diluted cDNA (3:20). The primer sequences for the miRNAs were available online by Quantabio (http://www.quantabio.com/products/microrna-profiling) and ordered from Metabion. Table 2 shows the sequences selected for each miRNA.

**Table 2:** Primer sequences of miRNAs selected for validation

miRNA	Primer sequence
hsa-miR-140-5p	CGCAGTGGTTTTACCCTATG
hsa-miR-191-5p	AACGGAATCCCAAAAGCAG
hsa-miR-214-3p	GCACAGCAGGCACAGACAG
hsa-let-7i-5p	CGTTCTGAGGTAGTAGTTTGTGCT
hsa-miR-103a-3p (control)	CAGCATTGTACAGGGCTATGAA

The amplification conditions included: denaturation at 95°C (10 min), amplification at 95°C (15 sec) and extension at 60°C (1 min) for 40 cycles using the 7500 Real-Time PCR System (*Applied Biosystems*). The miRNA, hsa-miR103a-3p was used as an endogenous control for all the reactions. In addition, non-enzyme controls (NEC) and non-template controls (NTC) were used where, in NEC, reverse transcriptase was not added during synthesis of cDNA and in NTC, no cDNA template was added, instead it was replaced by water. The RT-PCR were performed in triplicates for each sample.

### Data analysis

For the analysis of the RT-PCR data, normalized *C*_t_ values (Δ*C*_t_) were calculated for each sample according to the mean threshold of the miRNA of interest and the endogenous control. The Δ*C*_t_ of each miRNA in BB samples were compared with the Δ*C*_t_ of BA samples and Wilcoxon signed-rank test was performed where a *P*- value was considered significant at <0.05.

### Secondary phase: targeted genes for the validated miRNAs

#### Targeted genes

A total of 21 prediction tools, including BcmicrO (https://tools4mirs.org/software/target_prediction/bcmicro/) [33], BiTargeting (https://www.cs.bgu.ac.il/∼vaksler/BiTargeting.htm) [34], CoMeTa (http://cometa.tigem.it/) [35], Cupid (http://cupidtool.sourceforge.net/) [36], DIANA (http://diana.imis.athena-innovation.gr/DianaTools/index.php, ElMMo3 (http://www.mirz.unibas.ch/ElMMo3/index.php) [37], MBStar (https://www.isical.ac.in/∼bioinfo_miu/MBStar/MBStar_download20.htm) [38] microrna.org (http://www.microrna.org/microrna/home.do) [39], MirAncesTar (https://www.cs.mcgill.ca/∼blanchem/mirancestar/) [40,41], miRcode (http://www.mircode.org/) [42], mirCoX (http://210.212.254.116/mircox/pages/index.php) [43], miRDB (http://www.mirdb.org/) [44], MirMAP (https://mirmap.ezlab.org/) [45], MirTar (http://mirtar.mbc.nctu.edu.tw/human/) [46], miRTar2GO (http://www.mirtar2go.org/) [47], Mirza-G (http://www.clipz.unibas.ch/mirzag/) [48], MultiMiTar (https://www.isical.ac.in/∼bioinfo_miu/multimitar.htm) [49], PicTar (https://pictar.mdc-berlin.de/) [50], RepTar [51], RNAhybrid (https://bibiserv.cebitec.uni-bielefeld.de/rnahybrid) [52] and TargetRank (http://hollywood.mit.edu/targetrank/) [53], were used in order to identify predicted target genes for the miRNAs which showed significant differences between BB and BA samples. The genes selected were those detected to be predicted targets of two or more of the validated miRNAs. In order to identify the most promising target genes, a comprehensive literature search was performed in PubMed using specific key terms; ‘gene name’ and/or ‘aneurysm’, ‘cardiovascular disease’, ‘aorta’ and ‘miRNA of interest’. In addition, NCBI-Gene (https://www.ncbi.nlm.nih.gov/gene) and UniProt databases (https://www.uniprot.org/) were searched in order to identify any possible association of these proteins with aneurysms [54].

### cDNA synthesis and RT-PCR

Using the same 22 samples as with the miRNA validation phase, the expression levels of the mRNAs from the predicted nine target genes chosen were examined. Primers were designed based on the UTR of the mRNAs using TargetScan Human and Ensembl genome browser. cDNA synthesis and RT-PCR were performed using qScript microRNA cDNA Synthesis Kit (*Quanta Biosciences*) and EvaGreen master mix (ROX+), as described earlier. As an internal control, 18S rRNA was used. Table 3 shows the mRNA primer sequence for the target genes.

**Table 3:** mRNA primer sequence

Sequence name	Sequence
CCND2-Forward	AGTTATTGCTGGTGCAAAGA
CCND2-Reverse	GCCTCTAAGAAGTGGAGAGG
ABLIM1-Forward	AGCTCTGTCGAATTCATGG
ABLIM1-Reverse	CTTTGAGTCACACACACTCG
CRKL- Forward	TGTCTTAGTGTTTTAGTGACTAGGG
CRKL-Reverse	AGATGATGGATGAACCACAC
MTMR4-Forward	TTAGGGGAACAGTTCTAGGG
MTMR4-Reverse	TAGGAAAAGTCAGGTGCAAA
HEY1-Forward	TTGGTCTGTTTTTCTCCTTG
HEY1-Reverse	CAACGTTTTGCTCAGAAATA
FNDC5-Forward	ACAGGATAAAGGGCAGATGT
FNDC5-Reverse	TGCTCTAAGTGGATCAGAGG
NFIA-Forward	CATGCGACTTCAAGAAGGTTT
NFIA-Reverse	TGTGTCTTAATGCACTCTACTTTATCC
PPP1CB-Forward	TCATTTTCAGCACAGTGCAA
PPP1CB-Reverse	CAGTCAGGCTTCATATCAATTAGAC
TJP1-Forward	TCGTCACTGCTTAACTTCACA
TJP1-Reverse	TAGGCCAGGGCCATAGTAAA

### Data analysis

The expression levels of the mRNAs were analyzed according to their average Δ*C*_t_ as described earlier and they were further analyzed according to their fold-change expression differences between BB and BA samples. In cases were the fold-change was 1.5 or above, the difference was considered significant. Validated target genes of deregulated miRNAs were determined using MirTarbase (http://mirtarbase.mbc.nctu.edu.tw/php/index.php), which is a database of manually curated miRNA target gene interactions, obtained from the literature [55]. Data underlying the present study are available at the NCBI Gene Expression Omnibus (https://www.ncbi.nlm.nih.gov/geo/) under the accession number GSE151378.

## Results

### Primary phase: investigation of differentially expressed miRNAs between plasma samples

Using the gene expression profile obtained from the Nanostring data, a total of 80 miRNAs were detected in eight plasma samples and they were also confirmed in four tissue samples. The detection of the circulating levels of the miRNAs may be dependent on the diseased aortic tissue samples either by originating or being enriched in this particular tissue. Eight of these miRNAs, showed significant difference (*P*≤0.001) in their expression levels between BB and BA plasma samples and in all cases the expression was lower in BB samples while the levels were much higher in the tissue samples (Table 4).

**Table 4:** miRNAs showing significant difference between BB and BA samples

miRNA	Mean BB	Mean BA	*P*-value	Mean T
Hsa-let-7c-5p	0.22	5.47	0.007	1444.88
Hsa-let-7i-5p	0	3.30	0.002	748.18
Hsa-miR-140-5p	0.32	3.92	0.001	258.81
Hsa-miR-191-5p	0.18	2.89	0.021	498.97
Hsa-miR-214-3p	0	2.26	0.007	48.27
Hsa-miR-223-3p	0.64	15.71	0.001	96.89
Hsa-miR-23a-3p	0.98	8.83	0.015	12306.94
Hsa-miR-30b-5p	0	2.51	0.001	8.69

Plasma samples before (BB) and after (BA) repair surgery as well as the removed aortic sample (T).

he hsa-miR-223-3p presented the highest expression difference between BB and BA samples while the hsa-miR-191-5p, showed the lowest significance between the two sample types. For three, miRNAs (hsa-let-7i-5p, hsa-miR-214-3p and hsa-miR-30b-5p), the mean BB values were zero and this could be due to the extremely low expression, therefore they did not reach the threshold and could not be detected.

### miRNAs and ATAA pathways

In order to investigate any possible correlation between the eight miRNAs and ATAA pathways, the MirPath software was used. Three miRNAs (hsa-let-7i-5p, hsa-miR-140-5p, and hsa-miR-214-3p) were identified that significantly interact with relevant pathways, namely the Extracellular Matrix (ECM)–Receptor Interaction Pathway and one (hsa-miR-191-5p) with the Focal Adhesion Pathway (Table 5).

**Table 5:** miRNAs and predicted ATAA pathways

miRNAs	Pathway	*P*-value
Hsa-let-7i-5p	ECM–Receptor Interaction	5.16 e^-7^
Hsa-miR-140-5p	ECM–Receptor Interaction	0.00017
Hsa-miR-191-5p	Focal Adhesion	0.049
Hsa-miR-214-3p	ECM–Receptor Interaction	1.26 e^-14^

### miRNA validation

**Figure 1:**
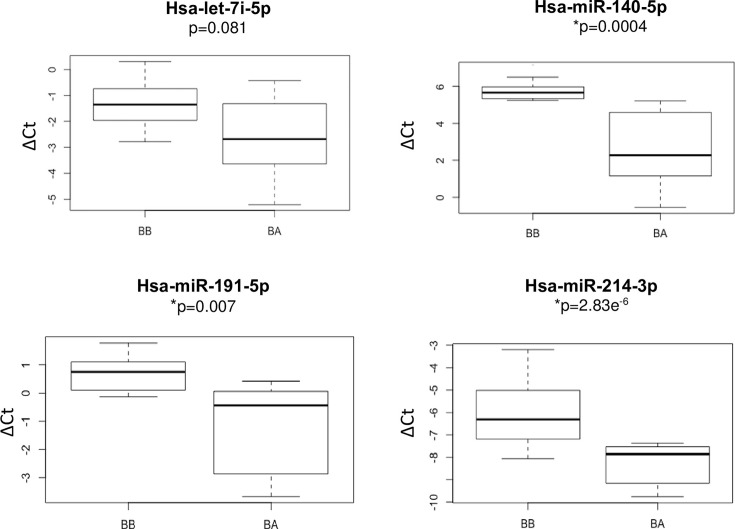
Differentially expressed plasma miRNAs from ATAA patients Relative levels of hsa-let-7i-5p, hsa-miR-140-5p, hsa-miR-191-5p, and hsa-miR-214-3p were validated in plasma samples from 11 patients collect before (BB) and after (BA) repair surgery. The following three, hsa-miR-140-5p (*P*-value = 0.0004), hsa-miR-191-5p (*P*-value = 0.007), and hsa-miR-214-3p (*P*-value = 2.83e^−6^) showed statistically significant differences. The expression threshold (*C*_t_) values of each miRNA were normalized to hsa-miR-103a-3p. *P<0.05; NS, non-significant.

**Figure 2:**
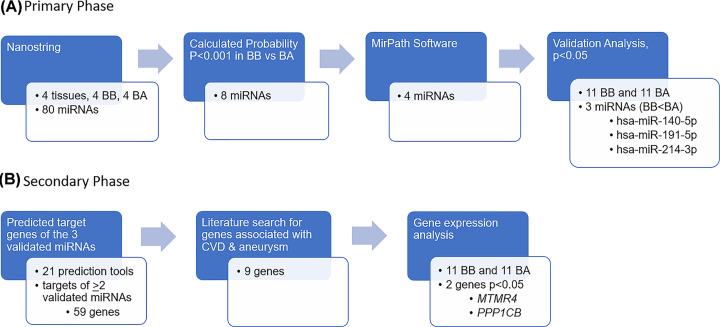
Summary of experimental design and analysis (**A**) Primary phase of experimental work which included the investigation of differentially expressed miRNAs between plasma samples from patients collected before (BB) and after (BA) repair surgery. (**B**) Secondary phase involving the investigation of the target genes for the validated miRNAs

The above four miRNAs chosen for validation in 22 plasma samples, showed lower expression in BB compared with BA samples, further supporting the results obtained using the nCounter miRNA Expression Assay. However, only hsa-miR-140-5p, hsa-miR-191-5p and hsa-miR-214-3p showed statistically different expression levels between the two plasma sample types with *P*-values, 0.0004, 0.007, and 2.83e^−6^, respectively (Figure 1). A flow chart summarizing the primary phase of the study is shown in Figure 2A.

### Secondary phase: targeted genes for the validated miRNAs

#### Targeted genes

The three miRNAs which were detected to be significantly different between BB and BA samples were further investigated using 21 prediction tools, identifying 59 genes as predicted targets for at least two of these three miRNAs. For this selection, at least 15 tools presented a consistent result for all miRNA-interacted target genes. After an in-depth literature search, which was performed for each of these genes, nine were selected since they were previously identified and could possibly be associated with CVDs including ATAAs (Table 6).

**Table 6:** The selected predicted target genes

Gene name	miRNA
*ABLIM1*	Hsa-miR-140-5p Hsa-miR-214-3p
*CCND2*	Hsa-mir-191-5p Hsa-miR-214-3p
*CRKL*	Hsa-mir-191-5p Hsa-miR-214-3p Hsa-miR-140-5p
*FNDC5*	Hsa-miR-140-5p Hsa-miR-214-3p
*HEY1*	Hsa-miR-140-5p Hsa-miR-191-5p
*MTMR4*	Hsa-miR-140-5p Hsa-miR-214-3p
*NFIA*	Hsa-miR-191-5p Hsa-miR-214-3p
*PPP1CB*	Hsa-miR-140-5p Hsa-miR-191-5p
*TJP1*	Hsa-miR-140-5p Hsa-miR-191-5p

Hsa-miR-140-5p is predicted to target seven (*ABLIM1, CRKL, FNDC5, HEY1, MTMR4, PPP1CB, TJP1*) of these genes and hsa-miR-191-5p (*CCND2, CRKL, HEY1, NFIA, PPP1CB*, TJP1) as well as hsa-miR-214-3p (*ABLIM1, CCND2, CRKL, FNDC5, MTMR4, NFIA*) to target six from the total of nine genes. *CRKL* is the only gene which is predicted to be targeted by all three validated miRNAs.

**Figure 3:**
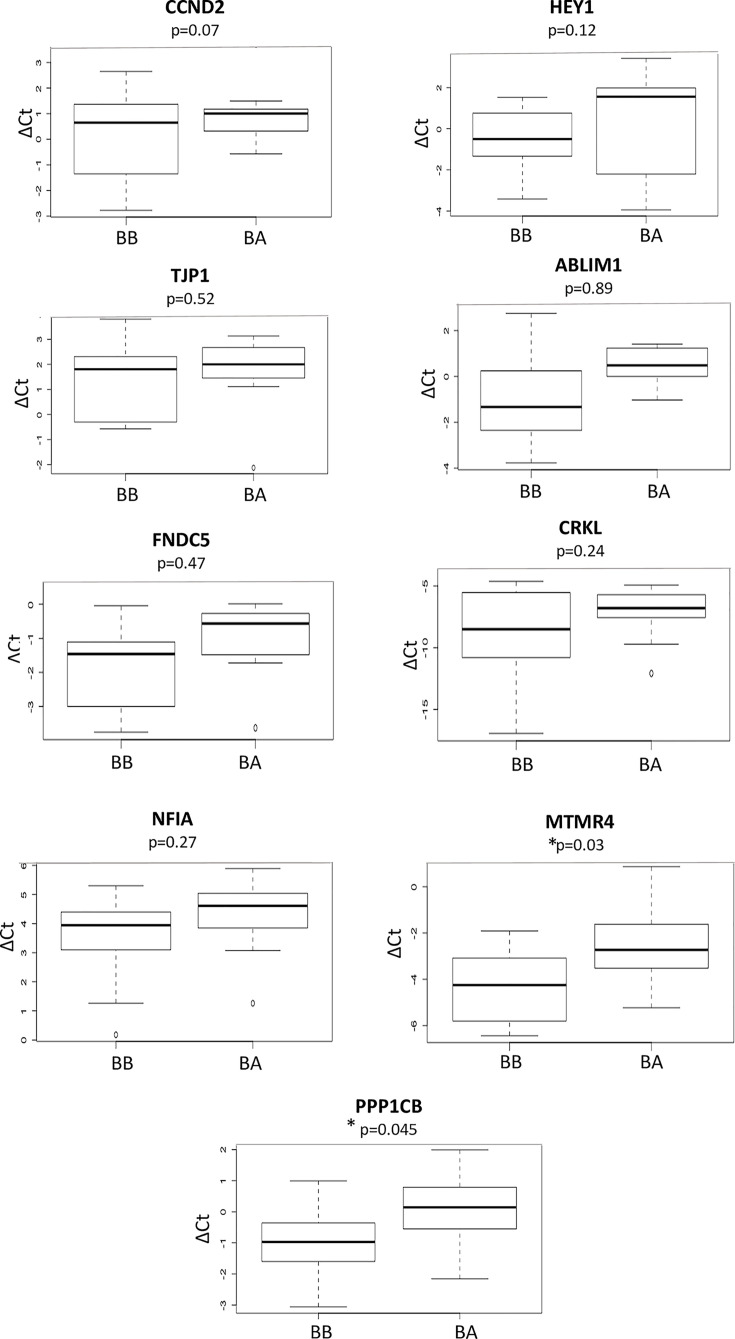
Differentially expressed genes in plasma from ATAA patients Average Δ*C*_t_ values of expressed genes (*ABLIM1, CCND2, CRKL, FNDC5, HEY1, MTMR4, NFIA, PPP1CB* and *TJP1*) in plasma samples from 11 patients collect before (BB) and after (BA) repair surgery. The values are lower in the BB compared with BA samples and so indicating higher expression levels in BB samples. The *MTMR4* and the *PPP1CB* reached a statistical significance.

Based on our results, all nine mRNAs examined (*CCND2, CRKL, HEY1, ABLIM1, FNDC5, TJP1, MTMR4, NFIA* and *PPP1CB*) showed higher expression in BB samples compared with BA samples with Myotubularin-related protein 4 (*MTMR4*) and Phosphatase 1 catalytic subunit β (*PPP1CB*) showing statistically significant differences with *P*-values 0.02 and 0.045, respectively (Figure 3). Further research using the miRTarBase showed strong experimental evidence associating *CCND2* with hsa-miR-191 and *CRKL* with hsa-miR-214-3p. Figure 2B represents a flow chart summarizing secondary phase of the study.

## Discussion

Although a number of miRNAs have been associated with ATAAs [18,19,23,56], no biomarker(s) exists for the non-invasive, early detection or screening for the disease.

In the present study, from a total of 80 miRNAs identified, eight (hsa-let-7c-5p, hsa-let-7i-5p, hsa-miR-140-5p, hsa-miR-191-5p, hsa-miR-214-3p, hsa-miR-223-3p, hsa-miR-23a-3p, hsa-miR-30b-5p), were identified with statistically significant different expression levels (*P*<0.05) between plasma samples collected before (BB) and after (BA) repair surgery from patients with ATAAs. From these eight miRNAs, four miRNAs, were selected based on their predicted interaction with pathways associated with TAAs, and were further validated in a larger sample size of 22 plasma samples showing the same pattern of expression, lower expression in BB compared with BA samples. However, only three (hsa-miR-140-5p, hsa-miR-190-5p and hsa-miR-214-3p) out of four miRNAs showed significant differences at the statistical level between BB and BA samples. According to the literature search performed by the authors, and to the best of their knowledge, as yet neither of these three miRNAs have been previously described as biomarkers associated with ATAAs.

These miRNAs have been previously described as potential biomarkers in a number of cardiovascular as well as other diseases. More specifically, hsa-miR-140-5p, was detected to be down-regulated in heart failure [57] as well as in carcinomas like Wilm’s tumor [58] and non-small-cell lung cancer [59] and overexpressed in children with obesity [60].

The expression levels of hsa-miR-214-3p were previously identified to be up-regulated in serum samples from patients with atrial fibrillation [61] whereas in patients with sinonasal inverted papilloma [62] and Parkinson [63] it was down-regulated. In addition, hsa-miR-191-5p was also down-regulated in numerous diseases including, acute myocardial infraction [64], Duchenne muscular dystrophy [65], renal cell carcinoma [66] and significantly overexpressed in attention-deficit/hyperactivity disorder [67]. Furthermore, a study by Licholai and colleagues (2016) identified [23] an overexpression of hsa-miR-191-5p in aneurysmal tissue when compared with normal aortic tissue. MiRNAs participate in various cell processes by targeting several genes that participate in a wide range of cell signaling pathways. More specifically, a single miRNA may affect expression of several target genes and may induce changes in various processes and pathways presenting additional mechanisms by which a disease may be induced [68,69]. Therefore, miRNAs are involved in many pathological processes that are present in different diseases and they may show a cell-specific regulation. The value of any suggested biomarker could be possible targeted therapeutics through the common pathways that are involved in the different diseases.

For the selection of genes that were targets of at least two out of the three validated miRNAs, a total of 59 were predicted as targets. A literature search investigated the possible association of these genes with CVD including aortic aneurysms. This was followed by gene expression analysis revealing that the mRNA levels of two genes (*MTMR4*, and *PPP1CB*) were statistically significant (*P*=0.03 and *P*=0.045, respectively) as well as being inversely correlated with the corresponding miRNA expression levels in the plasma samples.

MTMR4 (target for hsa-miR-140-5p and hsa-miR-214-3p) and PPP1CB (target for hsa-miR-140-5p and hsa-miR-191-5p) indicate a possible association with Transforming Growth Factor (TGF)-β signaling pathway, which in term plays a significant role in TAAs [70,71]. In particular, MTMR4 and PPP1C interact and de-phosphorylate SMAD2/3 complex and TGF-β receptor of this pathway, respectively and thus control its effects downstream. TGF-β signaling pathway targets and controls the activation of different genes [72], where a dysregulation or an overexpression of this pathway has been associated with syndromes known to cause aortic aneurysms [72–74].

Furthermore, it has been associated with matrix metalloproteinase (MMP) expression which plays a role in collagen degradation, (collagen being a major component of the aortic wall), suggesting that an overexpression of this pathway could lead to the up-regulation of MMPs and thus TAA formation [75–77]. The corresponding target genes for the validated miRNAs could be further investigated for any possible diagnostic and/or therapeutic value.

To further confirm these results, a larger validation study including more BB and BA samples should be performed to assess the sensitivity and specificity of the selected miRNAs that could be used as biomarkers for the early identification of the disease. In addition, the collection of plasma samples at least 1 month after the surgery could be more representative as normal-state condition compared with those samples that were collected 5–7 days post-surgically. Control plasma samples will need to be included in the extended study.

Overall, the three miRNAs described in this pilot study may prove to be promising biomarkers associated with ATAAs. More studies are warranted for further exploration of their contribution to ATAA pathogenesis and their potential role as clinical biomarkers.

## Supplementary Material

Supplementary Table S1

## Abbreviations

AD: aortic dissection
ATAA: ascending thoracic aortic aneurysm
BA: blood samples collected after surgery
BB: blood samples collected before surgery
CVD: cardiovascular disease
miRNA: microRNA
MTMR4: myotubularin-related protein 4
NEC: non-enzyme control
NTC: non-template control
PPP1CB: Phosphatase 1 catalytic subunit β
RT-PCR: real time polymerase chain reaction
T: tissue sample
TAA: thoracic aortic aneurysm
TGF-β: transforming growth factor-β
UTR: untranslated region
