# Supplementary material for: MicroRNAs in ascending thoracic aortic aneurysms
Source: Biosci Rep. 2020 Jul 27;40(7):BSR20200218. doi: 10.1042/BSR20200218 (PMC7385583; doi:10.1042/BSR20200218)
Supplement: Supplementary Table S1 [file BSR-2020-0218_supp.pdf]

Supplementary Table 1

Sample ID	Sample Type	260/280	260/230	Concentration (ng/µl)
148 BB 2.3	Plasma	1.45	0.53	33
148 BB 2.3	Plasma	1.53	0.61	11
162 BB 2.3	Plasma	1.38	0.50	8
162 BB 2.3	Plasma	1.61	0.27	3
206 BB 2.3	Plasma	1.45	0.58	13
206 BB 2.3	Plasma	1.44	0.55	22
224 BB 2.3	Plasma	1.47	0.62	5.5
224 BB 2.3	Plasma	1.43	0.64	20
148 T4	Tissue	2.03	1.95	65
162 T4	Tissue	2.1	1.71	82
206 T4	Tissue	2.03	1.94	73
224 T4	Tissue	2.05	1.80	52
